# A novel ecotype of *Anaplasma phagocytophilum* complex in questing *Ixodes fuscipes* ticks

**DOI:** 10.1186/s13071-025-07226-8

**Published:** 2026-02-09

**Authors:** María L. Félix, Adriana Santodomingo, Richard Thomas, Diego Queirolo, Sebastián Muñoz-Leal, José M. Venzal

**Affiliations:** 1 Laboratorio de Vectores y Enfermedades Transmitidas, Departamento de Ciencias Biológicas, CENUR Litoral Norte, Universidad de la República, Salto, Uruguay; 2 Centro de Investigación de Estudios Avanzados del Maule (CIEAM), Vicerrectoría de Investigación y Postgrado, Universidad Católica del Maule, Talca, Chile; 3 Departamento de Ciencia Animal, Facultad de Ciencias Veterinarias, Universidad de Concepción, Ñuble, Chillán, Chile; 4 Laboratorio de Ecología y Comportamiento de Fauna Silvestre, Departamento de Ciencias Biológicas, CENUR Litoral Norte, Universidad de la República, Salto, Uruguay; 5 Programa de Desarrollo de las Ciencias Básicas (PEDECIBA), MEC, UDELAR, Montevideo, Uruguay

**Keywords:** Tick-borne diseases, *Ixodes fuscipes*, *Anaplasma phagocytophilum*, Ecotypes, Uruguay

## Abstract

**Background:**

*Anaplasma phagocytophilum* is a complex of tick-borne bacteria of medical and veterinary relevance, whose eco-epidemiology is well characterized in the Northern Hemisphere but remains poorly understood in South America. Here, we report in Uruguay the detection and genetic characterization of a novel *A*. *phagocytophilum* ecotype in South America.

**Methods:**

Questing *Ixodes fuscipes,* the only member of the *Ixodes ricinus* complex in the country, were collected in five localities in Uruguay, and the presence of *Anaplasma* spp. DNA was assessed using PCR to amplify fragments of the 16S ribosomal RNA (*rrs*), *gltA* and *groEL* genes.

**Results:**

A total of 223 *Ixodes fuscipes* ticks were collected between 2017 and 2022 in five localities. PCR screening and subsequent sequencing identified *Anaplasma* spp. DNA in five nymphs from the Rivera and Tacuarembó departments. Phylogenetic analyses of *rrs*, *gltA* and *groEL* sequences of this bacteria confirmed the placement within the *A*. *phagocytophilum* clade. In particular, *groEL*-based phylogenies showed that Uruguayan sequences form a distinct and well-supported lineage, grouping with ecotype V (strain Patagonia) and being closely related to ecotype III. Pairwise genetic distance analyses of *groEL* sequences further supported the recognition of this lineage as a novel ecotype (ecotype VI). The detection of positive nymphs suggests acquisition from local vertebrate hosts, and the phylogenetic relationship among ecotypes III, V and VI, together with host records for immature and adult *I*. *fuscipes*, point to a potential role for small mammals, birds or cervids in maintaining the enzootic cycle of *A*. *phagocytophilum* strain “Uruguay.” Although vector competence of *I*. *fuscipes* remains to be determined, these findings provide preliminary evidence of the potential involvement of this tick species in local transmission and represent the second characterization of an *A*. *phagocytophilum* ecotype in South America.

**Conclusions:**

*Anaplasma phagocytophilum* is reported for the first time in Uruguay. The recognition of this new ecotype (VI) expands regional knowledge and underscores the influence of local host–vector assemblages in shaping *A*. *phagocytophilum* diversity. Broader host–vector surveys are needed to clarify its ecology, transmission dynamics and potential epidemiological implications in the region.

**Graphical Abstract:**

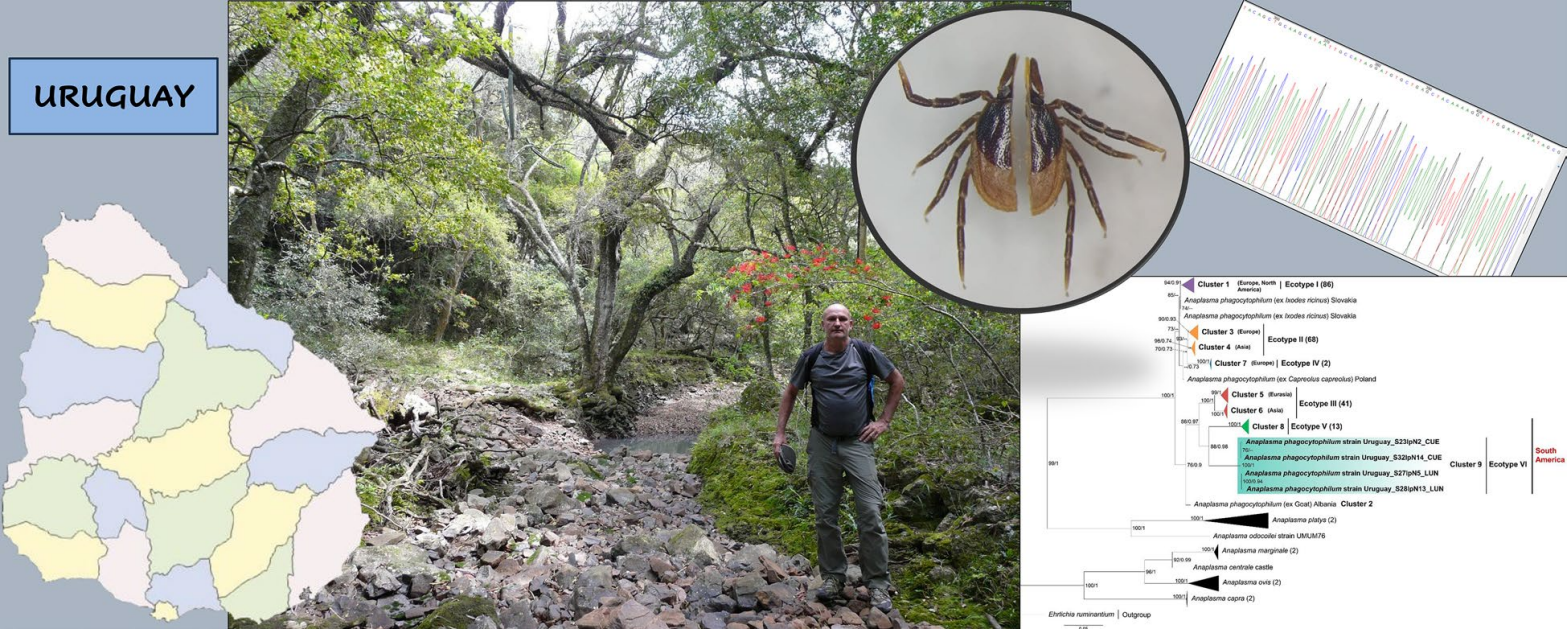

**Supplementary Information:**

The online version contains supplementary material available at 10.1186/s13071-025-07226-8.

## Background

The genus *Anaplasma* (*Rickettsiales*: *Anaplasmataceae*) comprises small, pleomorphic, Gram-negative bacteria that thrive in a variety of vertebrate hosts and which are primarily transmitted by ixodid ticks (Acari: Ixodidae) [1, 2]. The systematics of genus *Anaplasma* currently comprises six formally recognized species (*A. bovis*, *A. centrale*, *A. marginale*, *A. ovis*, *A. phagocytophilum* and *A. platys*), along with two provisional species (*A. odocoilei and A. capra*) and several additional candidate species and genovariants [3, 4].

The most widespread and genetically diverse species within genus *Anaplasma* is *A. phagocytophilum*, which poses a significant threat to human and animal health [3, 5]. This species is the etiological agent of human granulocytic anaplasmosis (HGA), tick-borne fever in domestic ruminants and granulocytic anaplasmosis in horses, dogs and cats [6]. Its natural enzootic cycles are well-described in the Northern Hemisphere, and its vertebrate hosts involve wild ruminants, rodents, insectivores, carnivores, raccoons, wild boards and, less frequently, birds and reptiles [3]. The primary vectors include the ticks *Ixodes ricinus* in Europe, *Ixodes persulcatus* in Eurasia and *Ixodes scapularis* and *Ixodes pacificus* in North America, where they have been recorded transmitting infections to humans and animals [5, 7, 8]. The ability of *A*. *phagocytophilum* to infect a broad spectrum of hosts points to its ecological plasticity and epidemiological significance [3].

Multiple strains and genovariants of *A*. *phagocytophilum* have been described, often linked to specific epidemiological cycles, host preferences and variable clinical pictures [9–11]. Molecular markers, such as the 16S ribosomal RNA (*rrs*), ankyrin A (*ankA*), major surface protein 4 (*msp4*), citrate synthase (*gltA*) and the heat-shock operon (*groEL*) genes, have been widely employed to disentangle this diversity [3, 4, 9, 12]. Among these, *groEL* stands out not only as the most informative marker for distinguishing *A. phagocytophilum* lineages but also as the locus with the most comprehensive sequence dataset, classified into four ecotypes that subdivide into seven phylogenetic clusters across Europe, Asia and North America [3, 4].

A bacterial ecotype is defined as a monophyletic group of strains sharing a similar ecological niche, for which between-ecotype sequence divergence is significantly greater than within-ecotype divergence for a given gene [13, 14]. In the case of *A*. *phagocytophilum*, ecotypes and phylogenetic clusters have been delineated based on *groEL* genetic divergence, geographic distribution, enzootic cycles, host range and pathogenic potential [3, 4, 9, 15]. For example, ecotypes I and II are strongly associated with *Ixodes* spp. ticks parasitizing cervids in Europe, ecotype III is linked to small mammals (rodents and insectivores) and their *Ixodes* spp. vectors in Eastern Europe and parts of Asia, whereas ecotype IV is associated with birds and their avian ticks, mainly in Europe [3]. Importantly, *A*. *phagocytophilum* strains belonging to ecotype I are zoonotic and include variants that cause HGA [3].

As key hosts in tick–pathogen systems, cervids sustain adult *Ixodes* spp. ticks, some of which can transmit *Anaplasma* spp., and they are also recognized as competent reservoir hosts for these infections [16]. In parallel, small mammals and birds contribute by feeding on larval and nymphal stages, thereby ensuring the continuity of *Ixodes* spp. life-cycles and the persistence of enzootic cycles of pathogens such as *Anaplasma* spp. Several *Anaplasma* spp. have been documented in South America [17], including the genetic detection and evidence of exposure to *A*. *phagocytophilum* in a variety of wild hosts [18–22]. However, ecotypes of this species were not described until in 2023, when Santodomingo et al. [4] characterized a novel ecotype of *A*. *phagocytophilum* (ecotype V) in wild pudu cervids (*Pudu puda*) and *Ixodes stilesi* ticks in Chile.

More recently, a study identified the occurrence of ecotype I in dogs in the Galapagos Islands, Ecuador [23]. Despite the current geographic restrictions associated with *A*. *phagocytophilum* ecotypes, these findings shed light on the epidemiology and systematics of the species beyond the Northern Hemisphere, revealing that South America may harbor a broader diversity of *A*. *phagocytophilum* ecotypes than previously recognized, with endemic lineages sustained by local host–vector assemblages. Indeed, South America presents ecological conditions capable of sustaining enzootic cycles of *Anaplasma* species, with its high diversity of *Ixodes* spp. ticks and wildlife hosts providing a natural setting favorable for their circulation and persistence [17, 24].

Uruguay has the environmental conditions, host–vector assemblages and an epidemiological context that make it a suitable setting for exploring *Anaplasma* species diversity [24, 25]. Records of three species (*A*. *platys*, *A*. *centrale*, and *A*. *marginale*) [26–28] and one lineage (*Anaplasma* sp. genotype Mazama) [25] highlight both the diversity already present and the need to further investigate hidden enzootic cycles involving other species, such as *A*. *phagocytophilum*. Accordingly, this study aimed to detect and characterize *Anaplasma* spp. using markers for ribosome- and protein-encoding genes, in questing *Ixodes fuscipes* ticks, the sole representative of the *Ixodes ricinus* complex in the country.

## Methods

### Study area

Sampling campaigns were conducted in five Uruguayan localities between September 2017 and May 2022. Four of these localities are situated in the northern region of the country: Arroyo Sepulturas, in Artigas Department (− 30.851389, − 56.072500); Lunarejo, in Rivera Department (− 31.141389, − 55.900278); Gruta de los Cuervos, in Tacuarembó Department (− 31.618889, − 56.046389); and Puntas de Arapey, in Salto Department (− 31.156944, − 56.140556). The fifth site, Laguna Negra, in Rocha Department (− 34.085833, − 53.738056), is found in the southeast of the country.

### Collection of samples

Ticks were collected on predetermined transects using the flagging method, which consists of dragging a white cloth measuring 1.20 × 0.80 m across the vegetation. Depending on the site, sampling covered an area of at least 750 to 1000 m^2^ and was conducted by two collectors. Ticks were picked up from the cloth every 5–10 m and preserved in tubes containing 95% ethanol (Sigma-Aldrich®, St. Louis, MO, USA) until laboratory processing [29, 30].

### Laboratory proceedings

#### Tick identification

Ticks were identified following the morphological descriptions and keys of Venzal et al. [31] and Nava et al. [24], using a Nikon SMZ1000 stereo microscope (Nikon Corp., Tokyo, Japan). To support morphological identification, we molecularly characterized one tick from each sampling site by sequencing fragments of the cytochrome* c* oxidase subunit I (*cox1*), amplified following Folmer et al. [32]. Primer sequences and PCR conditions are given in Table 1.

**Table 1:** Primers and thermal conditions used for PCR detection and genetic characterization of *Anaplasma phagocytophilum* and *Ixodes fuscipes*

Organism	Gene^a^	PCR	Primer	Sequence	*T*_o_ ©°)	Expected length (bp)	References
Ticks	*cox1*	Primary	LCO1490	GGTCAACAAATCATAAAGATATTGG	50	710	[32]
			HCO2198	TAAACTTCAGGGTGACCAAAAAATCA			
*Anaplasmataceae*	16S rRNA	Primary	EHR16SD	GGTACCYACAGAAGAAGTCC	55	345	[34]
			EHR16SR	TAGCACTCATCGTTTACA GC			
		Primary	fD1	AGAGTTTGATCCTGGCTCAG	55	~ 1500	[66]
			rP2	ACGGCTACCTTGTTACGACTT			
*Anaplasma* spp.	*groEl*	Primary	HS1a	AITGGGCTGGTAITGAAAT	48	~ 1400	[67, 68]
			HS6a	CCICCIGGIACIAIACCTTC			
		Nested	HS43	ATWGCWAARGAAGCATAGTC	55	1297	[69]
			HSVR	CTCAACAGCAGCTCTAGTAGC	55	1297	[69]
		Primary	EEgro1F	GAGTTCGACGGTAAGAAGTTCA	55	670	[70]
			Anagro712R	CCGCGATCAAACTGCATACC			
		Primary	Anagro122F	AAATACGGTWGTCACGGG	55	385	[71, 72]
			Anagro649R	CTTTCTTCRACAGTTATAAG			
		Primary	AnaGroe240F	ATTAGYAAGCCTTATGGGTC	55	432	
			AnaGro712R	CCGCGATCAAACTGCATACC			
		Primary	AnaplatF2	GCGTAGTCCGATTCTCC AGT	59	650	[71, 73]
			AnaGro712R	CCGCGATCAAACTGCATACC			
		Primary	gro607Fa	GAAGATGCWGTWGGWTGTACKGC	57	664	[74]
			gro1294Rb	AGMGCTTCWCCTTCWACRTCYTC			
		Nested	gro677Fb	ATTACTCAGAGTGCTTCTCARTG	55	315	[74]
			gro1121Rb	TGCATACCRTCAGTYTTTTCAAC			
		Primary	AnagroUruF	ATTAGYAAGCCTTATGGRTC	55	1072	This study
			groE-1236as	TCTTTRCGTTCYTTMACYTCAACTTC			[75]
*Anaplasma* spp.	*gltA*	Primary	EHR-CS131F	CAGGATTTATGTCTACTGCTGCTTG	54	1048	[76]
			EHR-CS1226R	CCAGTATATAAYTGACGWGGACG			
		Primary	F4b	CCAGGCTTTATGTCAACTGC	55	800	[77]
			R1b	CGATGACCAAAACCCAT			
		Nested	EHR-CS136F	TTYATGTCYACTGCTGCKTG	55	650	[77]
			EHR-CS778R	GCNCCMCCATGMGCTGG			

^a^*cox1* Cytochrome* c* oxidase subunit I, *gltA* citrate synthase, *groEL* heat-shock operon, *rrs* 16S ribosomal RNA

#### DNA isolation and* Anaplasmataceae* detection

Since the transmission of *A*. *phagocytophilum* relies mainly on horizontal transfer between ticks and vertebrate hosts, as well as on transstadial transmission in its vector [12], this study focused on nymphs and adult ticks.

Prior to DNA extraction, all collected ticks (kept in tubes containing 95% ethanol) were rinsed with distilled water for 10 min to remove ethanol and then sectioned longitudinally. Genomic DNA was isolated using the GeneJET Genomic DNA Purification Kit (Thermo Fisher Scientific Baltics, Vilnius, Lithuania), according to manufacturer’s instructions. DNA was eluted in 100 μl of buffer (10 mM Tris–HCl, pH 9.0, 0.1 mM EDTA), following which DNA concentration was quantified using a Nanodrop 2000 spectrophotometer (Thermo Fisher Scientific, Waltham, MA, USA) and the quality of the DNA sample was checked by assessing the A260/A280 absorbance ratio. Samples with absorbance ratios between 1.6 and 2.0 were accepted as suitable for PCR amplification protocols [33]. Extracted DNA from ticks was also tested by amplifying the *cox1* gene as an internal control.

*Anaplasma* spp. detection was achieved using primers EHR16SD/EHR16SR to amplify a short fragment of the *rrs* gene (short-*rrs*) [34]. For full genetic characterization, samples that tested positive in the screening assay underwent distinct PCR protocols targeting long fragments of the *rrs* (long-*rrs*), *gltA* and *groEL* genes, as detailed in Table 1. DNA of *A*. *marginale* isolate Uru 61 (GenBank accession number OP383022) was used as the positive control, and ultrapure water served as the no-template control. Each PCR was performed in 25 μl of reaction mixture (2 μl of each primer [0.4 μM], 4.5 μl of ultrapure water, 12.5 μl of MangoMix [Bioline, Memphis, TN, USA] and 4 μl of template DNA., in a SimpliAmp™ thermal cycler (Applied Biosystems, Thermo Fisher Scientific) 

PCR amplicons were verified by electrophoresis in a 1.5% agarose gel, loading 5 μl of product stained with GoodView Nucleic Acid Stain (Beijing SBS Genetech Co. Ltd., Beijing, China) and visualized with a CSLUVTSDUO312 ultraviolet (UV) transilluminator (Cleaver Scientific Ltd., Rugby, UK). Amplicons of the expected size were purified using the GeneJET PCR Purification Kit (Thermo Fisher Scientific Baltics) and subsequently submitted to Macrogen (Seoul, South Korea) for bidirectional Sanger sequencing.

### Genetic analysis

#### Assembly and sequence analyses

Consensus sequences were generated from AB1 files in Geneious Prime v2021.2.2 (www.geneious.com) using a Phred score threshold ≥ 20 during base calling [35, 36]. To identify orthologous sequences, we used the basic local alignment search tool (BLASTn) (https://blast.ncbi.nlm.nih.gov) to compare consensus sequences against the non-redundant GenBank nucleotide database (https://www.ncbi.nlm.nih.gov).

#### Phylogenetic analysis

Phylogenies were performed under maximum-likelihood (ML) [37] and Bayesian inference (BI) [38, 39] approaches, following the phylogenetic frameworks outlined by Santodomingo et al. [4] to resolve the evolutionary relationships of *Anaplasma* spp. based on long-*rrs*, *gltA* and *groEL* sequences, together with orthologous sequences identified through BLASTn searches. Alignments for each marker were constructed with the Multiple sequence alignment program MAFFT v7 algorithm using default parameters [40] and subsequently curated using the Block Mapping and Gathering with Entropy (BMGE) v1.1 under predefined parameters [41].

ML and BI trees were inferred in IQ-TREE v1.6.12 [42] and MrBayes v3.2.6 [43], respectively. Protein-encoding gene datasets (*gltA* and *groEL*) were partitioned into codon positions as described by Santodomingo et al. [4]. ML best-fitting substitution models and best-partition scheme for protein-encoding datasets were determined with ModelFinder command “TESTNEWONLYMERGE -mrate G” [44]. A non-protein-coding 16S rRNA gene best-fit evolutionary model was computed using the ModelFinder command “-m TESTNEWONLY -mrate G.” The reliability of the ML tree topologies was evaluated through hill-climbing strategies combined with a stochastic perturbation procedure and further supported by 1000 ultrafast bootstrap (UFBoot) replicates [45].

BI best evolutionary models and phylogenies were inferred using the MrBayes commands “lset nst = mixed rates = gamma” and “lset = mixed rates = invgamma” for non-coding and protein-encoding datasets, respectively [43, 46]. The robustness of the inferred BI tree was evaluated by sampling trees every 1000 generations, with the first 25% as burn-in, implementing four Markov chain Monte Carlo (MCMC) chains through two independent tests of 40 × 10^6^ generations. The correlation and effective sample size (ESS) of the MCMCs were confirmed using Tracer v1.7.1 [47]. All best-fit models were selected according to the Bayesian information criterion (BIC) [48]. Trees were visualized and edited with FigTree v1.4.1 (http://tree.bio.ed.ac.uk/software/figtree/) and Inkscape v1.3.1 (https://inkscape.org/es/). The final tree for each marker integrates the consensus topologies from ML and BI analyses, constructed as described by Santodomingo et al. [49].

#### Genetic distance analysis

Average sequence divergence within and among *A*. *phagocytophilum* ecotypes was assessed using the *groEL* sequences generated in the present study, in combination with the *groEL* dataset proposed by Santodomingo et al. [4], which comprises 214 sequences with > 70% coverage and uses *Anaplasma* *odocoilei* and *A*. *platys* as outgroups (218 sequences in total). The alignment was generated using the multiple sequence alignment program MAFFT with default settings. Subsequently, the corrected pairwise distance was assessed with raxmlGUI [50, 51] in RAxML v8 [52] with the GTR + GAMMA + I substitution model.

## Results

### Tick collection and identification

In total, 223 ticks were collected across five sampling localities, consisting of 19 adults (10 females and 9 males) and 204 nymphs (see Table 2 for details of tick life stage per locality). Morphological identification confirmed that all specimens correspond to *I*. *fuscipes*. DNA extractions yielded high-quality DNA, and PCR amplification of the tick *cox1* produced amplicons of the expected size (approx. 710 bp) in all samples, validating the success of the DNA extractions. *cox1* sequences obtained from one tick specimen per sampling site after BLASTn analyses showed 98.98–100% identity to *I. fuscipes* isolate IF URUI previously characterized in Uruguay, thus supporting the morphological identification of the specimens (Additional File 1: Table S1). Comparisons also showed 90.61–90.82% identity with *Ixodes pararicnus* and 91.65–92.26% with *Ixodes chacoensis* sequences, both members of the *I*. *ricinus* complex in the Southern Cone of South America.

**Table 2:** Metadata collected from *Ixodes fuscipes* ticks and samples that tested positive for *Anaplasma phagocytophilum*

Collection site (total no. of ticks)	Collection date	Developmental stage	Number of ticks	Positive samples (*n*) / code	GenBank accession number		
					*rrs*	*gltA*	*groEL*
Gruta de los Cuervos, Tacuarembó (88)	September 2017	Female	1	0			
		Nymph	7	0			
	December 2017	Nymph	10	0			
	January 2018	Nymph	3	1 / S23IpN2(42)	PX418211	PX394610	PX394611
	March 2018	Nymph	6	0			
	May 2018	Nymph	3	0			
	June 2018	Male	1	0			
		Nymph	9	0			
	July 2018	Female	1	0			
		Nymph	3	0			
	July 2021	Male	2	0			
		Female	3	0			
		Nymph	10	1 / S32IpN14(124)	PX418208	PX394607	PX394612
	September 2021	Nymph	1	0			
	November 2021	Male	1	0			
		Female	1	0			
		Nymph	1	0			
	February 2022	Nymph	5	0			
	May 2022	Male	1	0			
		Nymph	19	0			
Lunarejo, Rivera (106)	September 2017	Male	2	0			
		Nymph	7	0			
	November 2017	Nymph	8	0			
	December 2017	Nymph	5	0			
	March 2018	Nymph	1	0			
	May 2018	Nymph	7	0			
	June 2018	Male	1	0			
		Nymph	6	1 / S27IpN5(65)	PX418209	PX394609	PX394613
	July 2018	Nymph	13	1 / S28IpN13(90)	PX418212	PX394608	PX394614
	September 2021	Nymph	12	0			
	November 2021	Nymph	6	0			
	December 2021	Nymph	17	1 / S36IpN14(174)	PX418210	-	-
	February 2022	Nymph	3	0			
	May 2022	Nymph	18	0			
Arroyo Sepulturas, Artigas (11)	July 2021	Female	1	0			
		Nymph	4	0			
	October 2021	Female	1	0			
		Nymph	4	0			
	November 2021	Nymph	1	0			
Puntas de Arapey, Salto (2)	July 2021	Female	1	0			
		Male	1	0			
Laguna Negra, Rocha (16)	November 2020	Female	1	0			
		Nymph	15	0			
Total			223	5			

*gltA* Citrate synthase, *groEL* heat-shock operon, *rrs* 16S ribosomal RNA

### *Anaplasma* spp. detection and genetic analysis

Of the 223 ticks screened for *Anaplasma* spp., five ticks tested positive in the short-*rrs* PCRs, including two nymphs from Tacuarembó (2.27%) and three from Rivera (2.87%), producing amplicons of approximately 345 bp. Subsequent PCRs assays for full genetic characterization generated five sequences for long-*rrs* (742–1432 bp) and four sequences each for the *gltA* (579–628 bp) and *groEL* (983–1,035 bp) genes (Additional file 2: Table S2). BLASTn comparisons of the long-*rrs* genotypes revealed 98.16–99.80% identity with *A*. *phagocytophilum* sequences previously characterized in *Cervus elaphus* (OR268760) in the UK, a dog in South Africa (MK814402), a goat in China (HQ872464) and a human in South Korea (CP035303) (Additional file 2: Table S2). For *gltA*, pairwise analyses showed 85.52–86.08% identity with *A*. *phagocytophilum* isolate IS21 (OP585592) obtained from *I*. *stilesi* ticks in Chile (Additional file 2: Table S2). In contrast, *groEL* sequences displayed 93.43–93.59% identity with *A*. *phagocytophilum* isolate 21F-2 (MT018452) from *Marmota himalayana* in China (Additional file 2: Table S2).

### Phylogenetic and pairwise genetic analyses

**Fig. 1:**
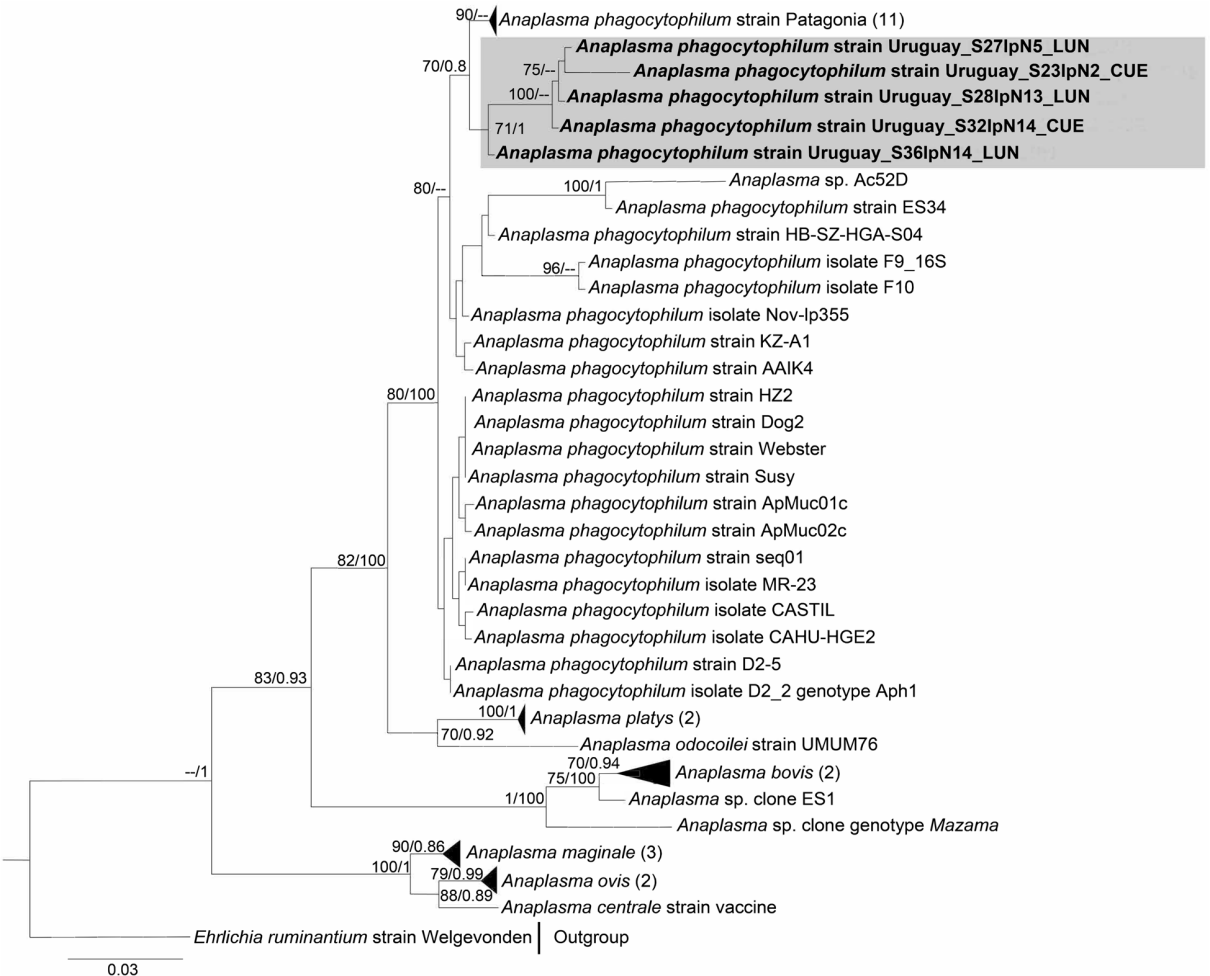
Maximum likelihood (ML) and Bayesian inference (BI) consensus tree of the long 16S ribosomal RNA gene (*rrs*) for *Anaplasma* species, highlighting the phylogenetic placement of the *Anaplasma phagocytophilum* “Uruguay” strain, shown within the gray box. Phylogenetic analyses incorporated 49 *Anaplasma* spp. sequences (alignment length: 1384 bp). Best-fit evolutionary models calculated for ML and BI methods were TPM3u + F + G4; and *M*_85_, *M*_134_, *M*_177_, *M*_179_, *M*_147_, *M*_200,_ respectively. Only nodes with ultrafast bootstrap values > 70% for ML [45] and Bayesian posterior probabilities ≥ 0.71 for BI [65] are displayed above or below branches. Numbers in brackets indicate the number of sequences in each collapsed clade. The scale bar represents nucleotide substitutions per site. Sequence details and GenBank accession numbers are provided in Additional file 3: Table S3

**Fig. 2:**
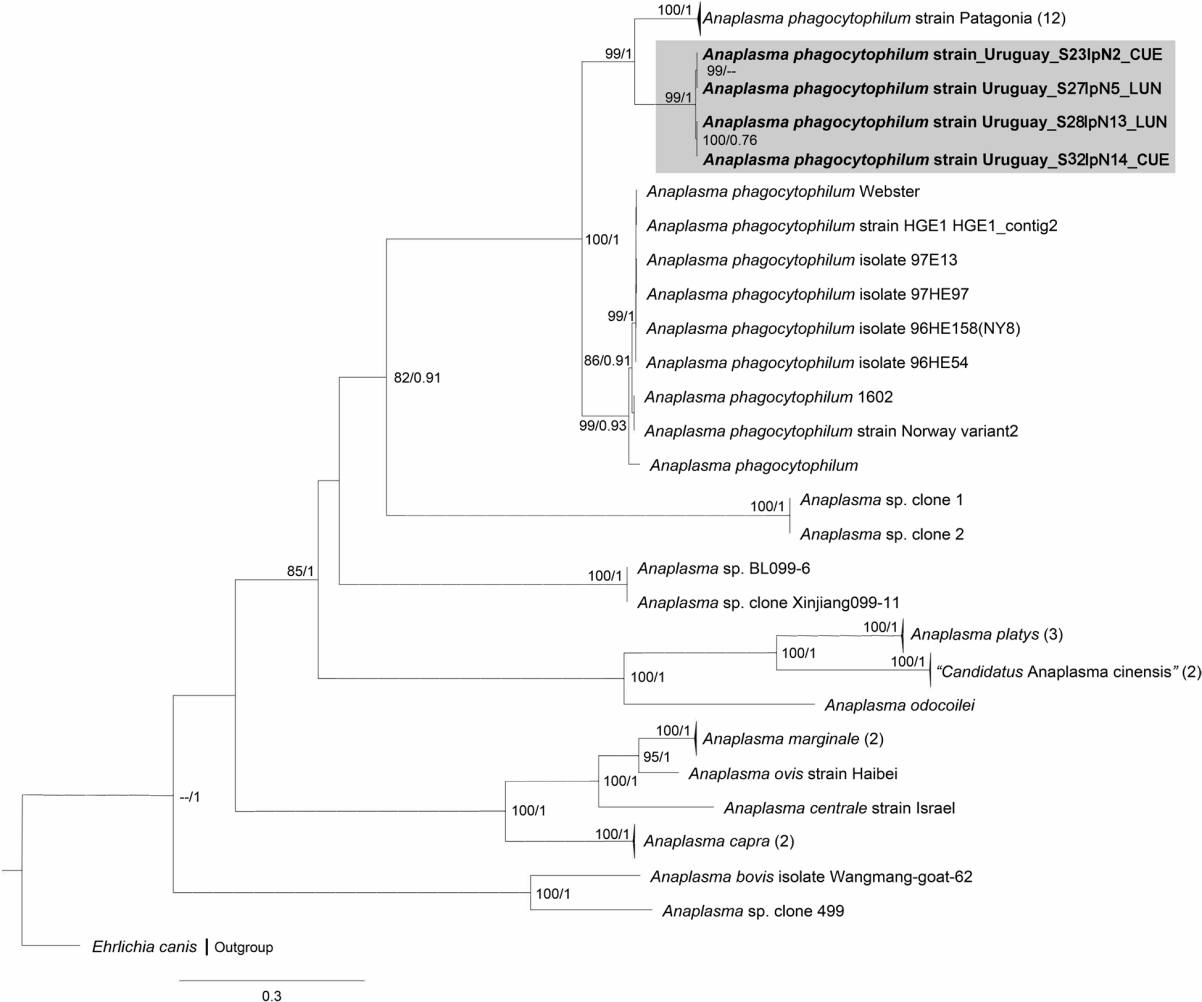
Maximum likelihood (ML) and Bayesian inference (BI) consensus tree of the citrate synthase gene (*gltA*) for *Anaplasma* species, highlighting the phylogenetic placement of the *Anaplasma phagocytophilum* “Uruguay” strain, shown within the gray box. Phylogenetic analyses incorporated 43 *Anaplasma* spp. sequences (alignment length: 1026 bp). Best-fit evolutionary models calculated for ML and BI methods were GTR + F + G4 (position-1), TIM3 + F + G4 (position-2), TPM2u + F + G4 (position-3); and *M*_64_, *M*_173_, *M*_175_, *M*_171_, *M*_125_ (position-1); *M*_80_, *M*_135_, *M*_164_, *M*_145_, *M*_166_ (position-2); *M*_90_, *M*_177_, *M*_152_, *M*_183_, *M*_136_ (position-3), respectively. Only nodes with ultrafast bootstrap values > 70% for ML [45] and Bayesian posterior probabilities ≥ 0.71 for BI [65] are displayed above or below branches. Numbers in brackets indicate the number of sequences in each collapsed clade. The scale bar represents nucleotide substitutions per site. Sequence details and GenBank accession numbers are provided in Additional file 3: Table S3

**Fig. 3:**
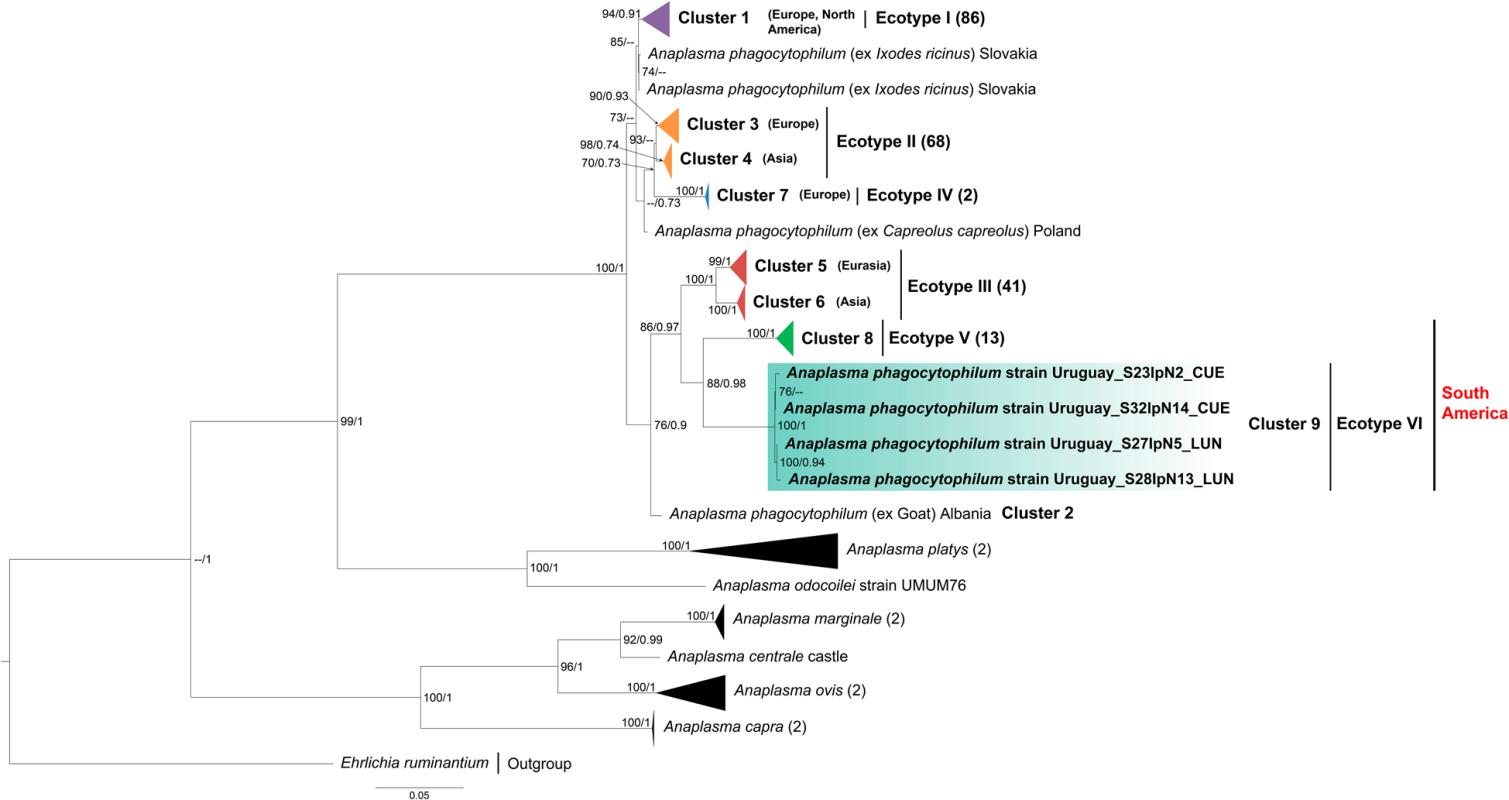
Maximum likelihood (ML) and Bayesian inference (BI) consensus tree of the heat-shock operon gene (*groEL*) for *Anaplasma* species, highlighting the phylogenetic placement of the *Anaplasma phagocytophilum* “Uruguay” strain, shown within the turquoise box. Phylogenetic analyses incorporated 230 *Anaplasma* spp. sequences (alignment length: 1224 bp). Best-fit evolutionary models calculated for ML and BI methods were TIM + F + G4 (position-1); TN + F + G4 (position-2); and K3Pu + F + G4 (position-3); and *M*_45_, *M*_136_, *M*_142_, *M*_130_, *M*_185_, *M*_139_ (position-1); *M*_81_, *M*_40_ (position-2); *M*_15_, *M*_50_, *M*_85_, *M*_122_ (position-3), respectively. Only nodes with ultrafast bootstrap values > 70% for ML [45] and Bayesian posterior probabilities ≥ 0.71 for BI [65] are displayed above or below branches. Ecotype colors (I–V) follow Jaarsma et al. [12] and Santodomingo et al. [4]. Numbers in brackets indicate the number of sequences in each collapsed clade. The scale bar represents nucleotide substitutions per site. Sequence details and GenBank accession numbers are provided in Additional file 4: Table S4

Phylogenies inferred from the three loci (long-*rrs*, *gltA*, and *groEL*) consistently placed *Anaplasma* spp. genotypes characterized from *I*. *fuscipes* ticks within the *A*. *phagocytophilum* clade, forming a monophyletic group with the *A*. *phagocytophilum* Patagonia strain (Figs. 1, 2, 3). For the *groEL* gene, the Uruguayan sequences formed a distinct, well-supported lineage (Cluster 9) together with *A*. *phagocytophilum* strain Patagonia, both closely related to ecotype III (Fig. 3). Cluster 9 was further supported by genetic distance analyses, as average sequence divergences within the Uruguayan cluster were consistently lower than those observed among ecotypes (Table 3).

**Table 3:** Average sequence divergence within ecotypes (highlighted in italics) and among ecotypes calculated according to the corrected pairwise distance for the *groEL* gene (alignment length: 1035 bp) of *Anaplasma phagocytophilum*

Ecotype	Ecotype I	Ecotype II	Ecotype III	Ecotype IV	Ecotype V	Ecotype VI
Ecotype I	*0.004930*					
Ecotype II	0.017587	*0.003021*				
Ecotype III	0.055914	0.050361	*0.002056*			
Ecotype IV	0.040154	0.030181	0.056496	*0.000001*		
Ecotype V	0.086059	0.077665	0.079001	0.078204	*0.003102*	
Ecotype VI	0.078110	0.078594	0.069003	0.079260	0.079696	*0.002135*

## Discussion

Based on phylogenies of the long-*rrs*, *gltA* and *groEL* genes, we identified an uncharacterized genovariant of *A*. *phagocytophilum* associated with *I*. *fuscipes* ticks, which we propose to designate as “Uruguay” variant (Figs. 1, 2, 3). Divergence values of partial *groEL* sequences further support its recognition as a novel ecotype within the *A*. *phagocytophilum* complex (ecotype VI) (Table 3), representing the first ecotype reported in Uruguay and the second ecotype characterized in South America. Notably, the *groEL* phylogeny provided strong evidence for regional differentiation, with the Uruguayan genovariant forming a distinct and well-supported lineage (Cluster 9) together with the strain Patagonia (ecotype V, Cluster 8) (Fig. 3). The detection of *A*. *phagocytophilum* in *I*. *fuscipes* aligns with its membership in the *I*. *ricinus* complex, a group that includes species such as *Ixodes scapularis* and *I*. *ricinus*, both well recognized for their involvement in *Anaplasma* spp. enzootic cycles in the Northern Hemisphere [3, 53].

The detection of ecotype VI in Uruguay, alongside ecotype V previously reported in pudu deers and *I. stilesi* ticks in Chile [4], supports the existence of multiple native *A. phagocytophilum* lineages circulating in the Southern Cone of South America. In contrast, the recent report of ecotype I in dogs in the Galápagos Islands [23] likely reflects an introduction rather than an natural endemic cycle. Collectively, these findings suggest that *A. phagocytophilum* is not restricted to recent introductions from the Northern Hemisphere but also includes native lineages that circulate in local wildlife-tick systems, maintained through distinct enzootic cycles under local ecological conditions. Ecotypes V and VI clearly contrast with their northern counterparts, differing not only phylogenetically (Fig. 3) and geographically but also in terms of the identity of their possible vectors and vertebrate hosts. These patterns point to the role of local host–vector assemblages in shaping *A. phagocytophilum* diversity in South America [4].

In the Northern Hemisphere, the eco-epidemiology of the main *A. phagocytophilum* ecotypes (I and II) is well characterized: cervids act as major reservoirs for both ecotypes while supporting adult *Ixodes* spp. ticks, whereas small mammals can also contribute to their maintenance by hosting larval and nymphal stages, thereby ensuring the continuity of enzootic cycles [3, 8]. Birds also play a role in maintaining immature stages of *Ixodes* spp., and are especially involved in the circulation of ecotype IV, which is associated with ornithophilic vectors such as *Ixodes frontalis* and *Ixodes ventalloi*; however, their contribution to the persistence of ecotypes I and II appears to be more limited and secondary [3].

Variants of *A. phagocytophilum* show host and vector specificity, leading to the maintenance of independent enzootic cycles that reflect adaptation to distinct ecological niches [3, 9]. From an ecological perspective, the recognition of “Uruguay” genovariant as an additional *A. phagocytophilum* ecotype associated with *I. fuscipes* points to a local transmission cycle in Uruguay, likely involving the vertebrates on which these ticks feed as potential reservoir hosts [3, 4]. Due to the close phylogenetic relationship between ecotype VI and ecotype V (Fig. 3), it cannot be ruled out that both lineages share similar eco-epidemiological strategies, including the involvement of deer in their maintenance. In this context, the Gray brocket deer (*Subulo gouazoubira*), the only cervid recorded as the main host of adult *I*. *fuscipes* [54], could be involved in the cycle of this new ecotype, although this hypothesis requires evaluation in future studies. It should be noted, however, that no adult ticks tested positive in this study, and given the small sample size (*n* = 19), broader sampling will be required to assess the potential role of this life stage in the enzootic cycle of ecotype VI.

Although *A. phagocytophilum* is primarily transmitted by *Ixodes* spp. [3], the mere detection of its DNA in *I*. *fuscipes* is insufficient evidence to assume a vectorial role [55]. Since *Ixodes* species feed on up to three different hosts during their life-cycle [56], the detected DNA could reflect residual, undigested blood of a previously infected host rather than an infection in the tick itself. Importantly, all ticks that tested positive in this study were nymphs collected from vegetation, and none of these showed signs of engorgement. Ultimately, confirmation of vector competence will require additional evidence, such as successful pathogen isolation, detection of *A. phagocytophilum* in salivary glands or experimental transmission to vertebrate hosts [55].

*Ixodes* species themselves have not been recognized as reservoirs of *A. phagocytophilum* [3], and transovarial transmission has only been documented in *Dermacentor albipictus* ticks [57]. The detection of positive nymphs in our study suggests that immature *I*. *fuscipes* likely acquire the bacterium from vertebrate hosts occurring in the localities of Lunarejo (Rivera Department) and Gruta de los Cuervos (Tacuarembó Department). Small mammals and birds are well established as primary hosts for larval and nymphal stages of *Ixodes* spp. [56], and in South American countries, immature *I*. *fuscipes* have been recorded parasitizing rodents such as *Akodon azarae* and *Oligoryzomys nigripes*, as well as birds, including *Phacellodomus striaticollis*, *Syndactyla rufosuperciliata*, *Turdus rufiventris* and *Turdus albicollis* [54].

Notably, ecotypes V (Patagonia) and VI (Uruguay) cluster together and are closely related to ecotype III (Fig. 3), which involves small mammals and their *Ixodes* spp. ticks in its enzootic cycles. This phylogenetic proximity suggests that the South American ecotypes (V and VI) may likewise rely on small mammals for their maintenance. Taken together, observed host associations, their overlapping distributions in Tacuarembó and Rivera Departments [54, 58, 59] and the phylogenetic link between ecotypes V, VI and III highlight plausible vertebrate hosts candidates for involvement in the transmission cycles of *A. phagocytophilum*. Future investigations should test this hypothesis through targeted sampling of both vertebrate hosts and *I*. *fuscipes* ticks to detect *A*. *phagocytophilum* in these and other regions where the species occurs. Meanwhile, the epidemiological cycle of *A*. *phagocytophilum* genovariant “Uruguay” remains undetermined.

Beyond Uruguay, several *rrs* genotypes associated with *A*. *phagocytophilum* have been reported in South American mammals, including deer (*S*. *gouazoubira*), rodents (*Cavia* sp. and *Calomys cerqueirai*), peccaries (*Tayassu pecari* and *Dicotyles tajacu*), sloths (*Bradypus tridactylus*) and coatis (*Nasua nasua*) [19–22], as well as in South American birds such as black vultures (*Coragyps atratus*), Orinoco geese (*Oressochen jubatus*), dusky-legged guans (*Penelope obscura*), caracaras (*Caracara plancus*) and Magellanic penguins (*Spheniscus magellanicus*) [60–63]. While these reports provide valuable preliminary insights, most detections relied on short *rrs* fragments (382–544 bp), lacked corresponding *gltA* or *groEL* sequences and were based on limited taxon sampling for phylogenetic analyses. As BLASTn comparisons did not reveal matches between the *rrs* sequences reported in those studies and the present study, and given that phylogenetic inferences in *Anaplasma* spp. require sufficiently long *rrs*, *gltA*, and *groEL* sequence fragments [3, 4, 64], complemented by dense taxon sampling of both ingroup and outgroup taxa to delimit lineages and evaluate their monophyly and evolutionary relationships with higher confidence [4], we did not include those sequences in our analyses.

Finally, this study has a number of limitations worth highlighting. First, the low number of positive nymphs (*n* = 5) restricts any evaluation of both the epidemiological significance of the findings (including bacterial prevalence and enzootic cycle stability) and the assessment of genetic variability within the detected lineage. Second, pathogen detection relied solely on DNA recovered from ticks, with functional evidence of infection or transmission lacking, which prevents the role of *I*. *fuscipes* as a vector. Third, the absence of vertebrate host analysis from surveyed areas limited our capacity to identify potential host reservoirs and to approximate the enzootic cycle. Consequently, the ecological and epidemiological interpretations presented here should be considered to be preliminary.

## Conclusions

The eco-epidemiology of *A*. *phagocytophilum* in South America remains less well understood than that in the Northern Hemisphere. Based on *groEL* divergence criteria typically applied for ecotype delimitation, this lineage shows genetic differentiation comparable to those observed among established ecotypes. In this context, our study documents a novel ecotype (ecotype VI, “Uruguay” genovariant) associated with *I*. *fuscipes* ticks, expanding regional diversity and advancing understanding of the pathogen’s ecology and systematics. Moreover, the close phylogenetic proximity of the Uruguayan lineage to ecotypes V and III, together with ecological evidence, raise the possibility that small mammals, in addition to cervids like *S*. *gouazoubira*, may contribute to its maintenance, a hypothesis that requires confirmation. While these data suggest a potentially novel ecotype, formal recognition requires further evidence from a broader sampling of ticks and vertebrate hosts. Overall, our findings shed light on the potential involvement of *I*. *fuscipes* ticks in local *A*. *phagocytophilum* enzootic cycles and highlight the necessity for broader, integrated host–vector investigations across South America. However, until further comprehensive studies have addressed expanded tick and vertebrate host sampling, vector competence, host range, transmission dynamics and pathogenicity, the ecological and epidemiological implications of the *A*. *phagocytophilum* “Uruguay” lineage remains unresolved.

## Supplementary Information

Additional file 1: Table S1. BLASTn matches of partial cytochrome* c* oxidase I (*cox1*) sequences retrieved from *Ixodes* *fuscipes* collected in Uruguay.

Additional file 2: Table S2. BLASTn comparisons of *Anaplasma phagocytophilum* sequences for each locus (16S rRNA, *gltA* and *groEL*).

Additional file 3: Table S3. GenBank accession numbers of *Anaplasma* spp. sequences used for phylogenic analyses based on 16S rRNA (*rrs*) and *gltA* genes. Sequences generated in this study are highlighted in bold.

Additional file 4: Table S4. GenBank accession numbers of the *Anaplasma* spp. sequences used for phylogenic analyses based on *groEL* gene. Sequences generated in this study are highlighted in bold.

## Data Availability

The data supporting the findings of the study are provided within the manuscript or supplementary information files.

## Abbreviations

16S rRNA: 16S Ribosomal ribonucleic acid
*ankA*: Ankyrin A
BI: Bayesian inference
BIC: Bayesian information criteria
BLASTn: Basic local alignment search tool
BMGE: Block Mapping and Gathering with Entropy
ESS: Effective sample size
*cox1*: Cytochrome* c* oxidase subunit I
*gltA*: Citrate synthase
*groEL*: Heat-shock operon
HGA: Human granulocytic anaplasmosis
MAFFT: Multiple sequence alignment program
MCMC: Markov chain Monte Carlo
*msp4*: Major surface protein 4
UFBoot: Ultrafast bootstrap
