# Supplementary material for: A novel ecotype of Anaplasma phagocytophilum complex in questing Ixodes fuscipes ticks
Source: Parasit Vectors. 2026 Feb 9;19:97. doi: 10.1186/s13071-025-07226-8 (PMC12927253; doi:10.1186/s13071-025-07226-8)
Supplement: Supplementary file 1 — Additional file 1: Table S1. BLASTn matches of partial cytochrome c oxidase I (cox1) sequences retrieved from Ixodes fuscipes collected in Uruguay. [file 13071_2025_7226_MOESM1_ESM.docx]

**Additional File 1: Supplementary Table S1.** BLASTn matches of partial cytochrome c oxidase I (*cox1*) sequences retrieved from *Ixodes* *fuscipes* collected in Uruguay.

| **Sequence length (bp)** | **Genotype** | **Accession number NCBI** | **BLAST subjects** | **Identity % (Identities)** | **Query cover (%)** | **E-value** | **Gaps** | **GenBank  accession number  of matching sequences** | **Country of origin  from matching  sequences** |
| --- | --- | --- | --- | --- | --- | --- | --- | --- | --- |
| 627 | *Ixodes fuscipes* isolate S39IpN37_Lunarejo | PX443922 | *Ixodes fuscipes* IF URUI | 100% (492/492) | 78% | 0 | 0/492 | OQ536433 | Uruguay |
| 627 | *Ixodes fuscipes* isolate S34IpH1_Sepulturas | PX443923 | *Ixodes fuscipes* IF URUI | 100% (492/492) | 78% | 0 | 0/492 | OQ536433 | Uruguay |
| 627 | *Ixodes fuscipes* isolate S32IpM21_Puntas_Arapey | PX443924 | *Ixodes fuscipes* IF URUI | 98.98% (487/492) | 78% | 0 | 0/492 | OQ536433 | Uruguay |
| 627 | *Ixodes fuscipes* isolate S31IpH1_Laguna_Negra | PX443925 | *Ixodes fuscipes* IF URUI | 98.98% (487/492) | 78% | 0 | 0/492 | OQ536433 | Uruguay |
| 627 | *Ixodes fuscipes* isolate S39IpM1_GrutadlCuervos | PX443926 | *Ixodes fuscipes* IF URUI | 98.98% (487/492) | 78% | 0 | 0/492 | OQ536433 | Uruguay |
