# Supplementary material for: A novel ecotype of Anaplasma phagocytophilum complex in questing Ixodes fuscipes ticks
Source: Parasit Vectors. 2026 Feb 9;19:97. doi: 10.1186/s13071-025-07226-8 (PMC12927253; doi:10.1186/s13071-025-07226-8)
Supplement: Supplementary file 3 — Additional file 3: Table S3. GenBank accession numbers of Anaplasma spp. sequences used for phylogenic analyses based on 16S rRNA (rrs) and gltA genes. Sequences generated in this study are highlighted in bold. [file 13071_2025_7226_MOESM3_ESM.docx]

**Additional file 3: Table S3.** GenBank accession numbers of *Anaplasma* spp. sequences used for phylogenic analyses based on 16S rRNA (*rrs*) and *gltA* genes. Sequences generated in this study are highlighted in bold.

| **Sequence name** | **GenBank  accession number** | **Host** | **Country** |
| --- | --- | --- | --- |
| **16S rRNA** | | | |
| *Anaplasma phagocytophilum* strain HZ2 | CP006616 | Human | USA |
| *Anaplasma phagocytophilum* strain HB-SZ-HGA-S04 | HQ872464 | Goat | China |
| *Anaplasma phagocytophilum* strain KZ-A1 | CP035303 | Human | South Korea |
| *Anaplasma phagocytophilum* strain seq01 | OR268760 | *Cervus elaphus* | United Kingdom |
| *Anaplasma phagocytophilum* strain D2-5 | MK814402 | Dog | South Africa |
| *Anaplasma phagocytophilum* strain Webster | NR044762 | Human | USA |
| *Anaplasma phagocytophilum* strain Dog2 | CP006618 | Dog | USA |
| *Anaplasma phagocytophilum* strain Susy | AY527213 | Horse | Sweden |
| *Anaplasma phagocytophilum* strain ApMuc02c | JX173652 | *Ixodes ricinus* | Australia |
| *Anaplasma phagocytophilum* isolate D2_2 genotype Aph1 | MK814406 | Dog | South Africa |
| *Anaplasma phagocytophilum* strain ApMuc01c | JX173651 | Dog | Germany |
| *Anaplasma phagocytophilum* isolate F9_16S | MW677507 | *Ixodes tapirus* | Panama |
| *Anaplasma phagocytophilum* isolate F10_16S | MW677508 | *Ixodes tapirus* | Panama |
| *Anaplasma phagocytophilum* isolate CAHU-HGE2 | AF093789 | Human | USA |
| *Anaplasma phagocytophilum* isolate MR-23 | KP276588 | *Ixodes pacificus* | USA |
| *Anaplasma phagocytophilum* isolate CASTIL | AF172166 | Horse | USA |
| *Anaplasma phagocytophilum* strain ES34 | AB196720 | *Cervus nippon yesoensis* | Japan |
| *Anaplasma* sp. Ac52D | AB588974 | Deer | Japan |
| *Anaplasma phagocytophilum* strain AAIK4 | KR611719 | *Apodemus agrarius* | South Korea |
| *Anaplasma phagocytophilum* isolate Nov-Ip355 | HM366580 | *Ixodes persulcatus* | Russia |
| *Anaplasma phagocytophilum* strain Patagonia 36S | OP579238 | *Pudu puda* | Chile |
| *Anaplasma phagocytophilum* strain Patagonia 45S | OP579239 | *Pudu puda* | Chile |
| *Anaplasma phagocytophilum* strain Patagonia 5P | OP579240 | *Pudu puda* | Chile |
| *Anaplasma phagocytophilum* strain Patagonia 8P | OP579242 | *Pudu puda* | Chile |
| *Anaplasma phagocytophilum* strain Patagonia 7P | OP579241 | *Pudu puda* | Chile |
| *Anaplasma phagocytophilum* strain Patagonia IS25 | OP579248 | *Ixodes stilesi* | Chile |
| *Anaplasma phagocytophilum* strain Patagonia IS19 | OP579246 | *Ixodes stilesi* | Chile |
| *Anaplasma phagocytophilum* strain Patagonia IS22 | OP579247 | *Ixodes stilesi* | Chile |
| *Anaplasma phagocytophilum* strain Patagonia 24P | OP579243 | *Pudu puda* | Chile |
| *Anaplasma phagocytophilum* strain Patagonia IS18 | OP579245 | *Ixodes stilesi* | Chile |
| *Anaplasma phagocytophilum* strain Patagonia IS17 | OP579244 | *Ixodes stilesi* | Chile |
| ***Anaplasma phagocytophilum* strain Uruguay_S27IpN5_LUN** | **PX418209** | ***Ixodes fuscipes*** | **Uruguay** |
| ***Anaplasma phagocytophilum* strain Uruguay_S23IpN2_CUE** | **PX418211** | ***Ixodes fuscipes*** | **Uruguay** |
| ***Anaplasma phagocytophilum* strain Uruguay_S28IpN13_LUN** | **PX418212** | ***Ixodes fuscipes*** | **Uruguay** |
| ***Anaplasma phagocytophilum* strain Uruguay_S32IpN14_CUE** | **PX418208** | ***Ixodes fuscipes*** | **Uruguay** |
| ***Anaplasma phagocytophilum* strain Uruguay_S36IpN14_LUN** | **PX418210** | ***Ixodes fuscipes*** | **Uruguay** |
| *Anaplasma platys* | LC269820 | Dog | Zambia |
| *Anaplasma platys* | AY530806 | Dog | Spain |
| *Anaplasma odocoilei* strain UMUM76 | JX876644 | *Odocoileus virginianus* | USA |
| *Anaplasma bovis* | AB196475 | *Haemaphysalis longicornis* | Japan |
| *Anaplasma bovis* | U03775 | Bovine | Senegal |
| *Anaplasma* sp. clone ES1 | KC811530 | *Elephantulus myurus* | South Africa |
| *Anaplasma* sp. clone genotype Mazama | MN817942 | *Mazama gouazoubira* | Uruguay |
| *Anaplasma ovis* isolate OVI | AF414870 | Goat | South Africa |
| *Anaplasma ovis* | AJ633049 | Goat | China |
| *Anaplasma centrale* strain vaccine | AF414868 |  | South Africa |
| *Anaplasma marginale* isolate Lushi | AJ633048 | Cattle | China |
| *Anaplasma marginale* | AF311303 | Bovine | USA |
| *Anaplasma marginale* from Uruguay | AF414877 |  | Uruguay |
| *Ehrlichia ruminantium* strain Welgevonden | NR074155 |  | South Africa |
| ***gltA*** | | | |
| *Anaplasma phagocytophilum* strain Patagonia IS17 | OP585594 | *Ixodes stilesi* | Chile |
| *Anaplasma phagocytophilum* strain Patagonia IS25 | OP585602 | *Ixodes stilesi* | Chile |
| *Anaplasma phagocytophilum* strain Patagonia IS19 | OP585593 | *Ixodes stilesi* | Chile |
| *Anaplasma phagocytophilum* strain Patagonia 36S | OP585597 | *Pudu puda* | Chile |
| *Anaplasma phagocytophilum* strain Patagonia 7P | OP585599 | *Pudu puda* | Chile |
| *Anaplasma phagocytophilum* strain Patagonia IS20 | OP585600 | *Ixodes stilesi* | Chile |
| *Anaplasma phagocytophilum* strain Patagonia IS18 | OP585598 | *Ixodes stilesi* | Chile |
| *Anaplasma phagocytophilum* strain Patagonia IS22 | OP585601 | *Ixodes stilesi* | Chile |
| *Anaplasma phagocytophilum* strain Patagonia 8P | OP585596 | *Pudu puda* | Chile |
| *Anaplasma phagocytophilum* strain Patagonia 24P | OP585595 | *Pudu puda* | Chile |
| *Anaplasma phagocytophilum* strain Patagonia IS21 | OP585592 | *Ixodes stilesi* | Chile |
| *Anaplasma phagocytophilum* strain Patagonia 5P | OP585591 | *Pudu puda* | Chile |
| ***Anaplasma phagocytophilum* strain Uruguay_S23IpN2_CUE** | **PX394610** | ***Ixodes fuscipes*** | **Uruguay** |
| ***Anaplasma phagocytophilum* strain Uruguay_S27IpN5_LUN** | **PX394609** | ***Ixodes fuscipes*** | **Uruguay** |
| ***Anaplasma phagocytophilum* strain Uruguay_S28IpN13_LUN** | **PX394608** | ***Ixodes fuscipes*** | **Uruguay** |
| ***Anaplasma phagocytophilum* strain Uruguay_S32IpN14_CUE** | **PX394607** | ***Ixodes fuscipes*** | **Uruguay** |
| *Anaplasma phagocytophilum* isolate 96HE158(NY8) | AY464138 |  | USA |
| *Anaplasma phagocytophilum* Webster | AF304136 |  | USA |
| *Anaplasma phagocytophilum* isolate 96HE54 | AY464136 |  | Japan |
| *Anaplasma phagocytophilum* isolate 97E13 | AY464134 |  | USA |
| *Anaplasma phagocytophilum* isolate 97HE97 | AY464137 |  | USA |
| *Anaplasma phagocytophilum* strain HGE1 HGE1_contig2 | APHH01000002 | Human | USA |
| *Anaplasma phagocytophilum* 1602 | AF304138 | Sheep | Spain |
| *Anaplasma phagocytophilum* strain Norway variant2 | CP015376 | Sheep | Norway |
| *Anaplasma phagocytophilum* | AY339602 | *Ixodes persulcatus* | Russian |
| *Anaplasma* sp. clone 1 | JN055361 | *Cervus nippon yasoensis* | Japan |
| *Anaplasma* sp. clone 2 | JN055362 | *Cervus nippon yasoensis* | Japan |
| *Anaplasma* sp. BL099-6 | KJ410280 | *Hyalomma asiaticum* | China |
| *Anaplasma* sp. clone Xinjiang099-11 | JX402608 | *Hyalomma asiaticum* | China |
| *Anaplasma platys* | AY077620 | Dog | Japan |
| *Anaplasma platys* | KR011928 | *Rhipicephalus* sp. | China |
| *Anaplasma platys* | EU516387 | Dog | Brazil |
| "*Candidatus* Anaplasma cinensis" AK-Rm-403 | MH716422 | *Rhipicephalus microplus* | China |
| "*Candidatus* Anaplasma cinensis" AK-Rm-228 | MH716426 | *Rhipicephalus microplus* | China |
| *Anaplasma odocoilei* | DQ020101 | *Odocoileus virginianus* | USA |
| *Anaplasma bovis* isolate Wangmang-goat-62 | MH255920 | Goat | China |
| *Anaplasma* sp. clone 499 | JN588561 | *Procyon lotor* | Japan |
| *Anaplasma marginale* | KX987367 | *Rhipicephalus microplus* | China |
| *Anaplasma marginale* | AF304140 |  | USA |
| *Anaplasma ovis* strain Haibei | CP015994 |  | China |
| *Anaplasma centrale* strain Israel | CP001759 |  | Israel |
| *Anaplasma capra* Zhengxiaocun-50 | MG869310 | Goat | China |
| *Anaplasma capra* AK-Rm-429 | MH716413 | *Rhipicephalus microplus* | China |
| *Ehrlichia canis* | AY647155 |  | Italy |
