# Supplementary material for: A novel ecotype of Anaplasma phagocytophilum complex in questing Ixodes fuscipes ticks
Source: Parasit Vectors. 2026 Feb 9;19:97. doi: 10.1186/s13071-025-07226-8 (PMC12927253; doi:10.1186/s13071-025-07226-8)
Supplement: Supplementary file 4 — Additional file 4: Table S4. GenBank accession numbers of the Anaplasma spp. sequences used for phylogenic analyses based on groEL gene. Sequences generated in this study are highlighted in bold. [file 13071_2025_7226_MOESM4_ESM.docx]

**Additional file 4: Table S4.** GenBank accession numbers of the *Anaplasma* spp. sequences used for phylogenic analyses based on *groEL* gene. Sequences generated in this study are highlighted in bold.

| **Sequence name** | **GenBank accession numbers** | **Host** | **Country** | **Cluster** | **Ecotype** |
| --- | --- | --- | --- | --- | --- |
| *Anaplasma phagocytophilum* isolate Omsk-6_Mglar | MN701631 | *Myodes glareolus* | Rusia | 5 | III |
| *Anaplasma phagocytophilum* isolate Omsk-104_Mrut | MN701630 | *Myodes rutilus* | Rusia | 5 | III |
| *Anaplasma phagocytophilum* isolate Omsk-65_Itr_m | MN701644 | *Ixodes trianguliceps* | Rusia | 5 | III |
| *Anaplasma phagocytophilum* isolate Omsk-Tr7 | KF745746 | *Ixodes trianguliceps* | Rusia | 5 | III |
| *Anaplasma phagocytophilum* isolate Omsk-Tr3 | KF745745 | *Ixodes trianguliceps* | Rusia | 5 | III |
| *Anaplasma phagocytophilum* clone Omsk-vole54 | KC583431 | *Myodes rufocanus* | Rusia | 5 | III |
| *Anaplasma phagocytophilum* isolate Omsk-41_Mrut | MN701636 | *Myodes rutilus* | Rusia | 5 | III |
| *Anaplasma phagocytophilum* isolate Omsk-56_Mruf | MN701635 | *Myodes rufocanus* | Rusia | 5 | III |
| *Anaplasma phagocytophilum* isolate Omsk-28_Apagr | MN701632 | *Apodemus agrarius* | Rusia | 5 | III |
| *Anaplasma phagocytophilum* isolate Omsk-Tr17 | KF745747 | *Ixodes trianguliceps* | Rusia | 5 | III |
| *Anaplasma phagocytophilum* clone Omsk-vole121 | KC583432 | *Myodes rutilus* | Rusia | 5 | III |
| *Anaplasma phagocytophilum* isolate 224691FITAFmouse | KF383236 | *Ixodes trianguliceps* | Slovakia | 5 | III |
| *Anaplasma phagocytophilum* isolate 228141LITMGvole | KF383232 | *Ixodes trianguliceps* | Slovakia | 5 | III |
| *Anaplasma phagocytophilum* isolate 227781LITMGvole | KF383235 | *Ixodes trianguliceps* | Slovakia | 5 | III |
| *Anaplasma phagocytophilum* isolate Omsk-51_Mruf | MN701629 | *Myodes rufocanus* | Rusia | 5 | III |
| *Anaplasma phagocytophilum* isolate Omsk-23_Mrut | MN701628 | *Myodes rutilus* | Rusia | 5 | III |
| *Anaplasma phagocytophilum* isolate Omsk-167_Micagr | MN701633 | *Microtus agrestis* | Rusia | 5 | III |
| *Anaplasma phagocytophilum* isolate 220166sMGvole | KF383231 | *Myodes glareolus* | Slovakia | 5 | III |
| *Anaplasma phagocytophilum* isolate Sv-shrew70 | HQ630617 | *Sorex araneus* | Rusia | 5 | III |
| *Anaplasma phagocytophilum* isolate Sv-vole8 | HQ630616 | *Myodes rutilus* | Rusia | 5 | III |
| *Anaplasma phagocytophilum* strain ItalyHU148 | KF031390 | *Myodes glareolus* | Italy | 5 | III |
| *Anaplasma phagocytophilum* isolate 227841LITMGvole | KF383233 | *Ixodes trianguliceps* | Slovakia | 5 | III |
| *Anaplasma phagocytophilum* isolate 21F-2 | MT018452 | *Marmota himalayana* | China | 5 | III |
| *Anaplasma phagocytophilum* isolate Omsk-17_Mrut | MN701639 | *Myodes rutilus* | Rusia | 6 | III |
| *Anaplasma phagocytophilum* isolate Omsk-9-13_Mruf | MN609907 | *Myodes rufocanus* | Rusia | 6 | III |
| *Anaplasma phagocytophilum* isolate RUS/Alt14-1625-Ipv | KX980041 | *Ixodes pavlovskyi* | Rusia | 6 | III |
| *Anaplasma phagocytophilum* isolate Nov-Ip456 | HM366570 | *Ixodes persulcatus* | Rusia | 6 | III |
| *Anaplasma phagocytophilum* isolate Irk-Ip625 | HM366571 | *Ixodes persulcatus* | Rusia | 6 | III |
| *Anaplasma phagocytophilum* isolate Nov-vole144 | HQ630614 | *Myodes rufocanus* | Rusia | 6 | III |
| *Anaplasma phagocytophilum* isolate Kh-vole305 | HQ630615 | *Myodes rufocanus* | Rusia | 6 | III |
| *Anaplasma phagocytophilum* isolate Tomsk-Ipr1 | KF701460 | *Ixodes trianguliceps* | Rusia | 6 | III |
| *Anaplasma phagocytophilum* clone Tuva-Ip2947 | KC753764 | *Ixodes persulcatus* | Rusia | 6 | III |
| *Anaplasma phagocytophilum* isolate Tomsk Dr-1 | KY379956 | *Dermacentor reticulatus* | Rusia | 6 | III |
| *Anaplasma phagocytophilum* isolate Tomsk-Ipr2 | KF701461 | *Ixodes persulcatus* | Rusia | 6 | III |
| *Anaplasma phagocytophilum* isolate Tomsk-Ipr3 | KF701462 | *Ixodes persulcatus* | Rusia | 6 | III |
| *Anaplasma phagocytophilum* isolate Tomsk-Ipr4 | KY684729 | *Ixodes persulcatus* | Rusia | 6 | III |
| *Anaplasma phagocytophilum* isolate Tomsk-Ipr6 | KY684731 | *Ixodes persulcatus* | Rusia | 6 | III |
| *Anaplasma phagocytophilum* isolate Tomsk-Ipr8 | KY684733 | *Ixodes persulcatus* | Rusia | 6 | III |
| *Anaplasma phagocytophilum* isolate Tomsk-Ipr5 | KY684730 | *Ixodes persulcatus* | Rusia | 6 | III |
| *Anaplasma phagocytophilum* isolate Tomsk-Ipr7 | KY684732 | *Ixodes persulcatus* | Rusia | 6 | III |
| *Anaplasma phagocytophilum* isolate Omsk-43_Mglar | MN701638 | *Myodes glareolus* | Rusia | 6 | III |
| *Anaplasma phagocytophilum* isolate RUS/Alt14-2442-Ipr/Ipv | MG182152 | *Ixodes persulcatus* | Rusia | 6 | III |
| *Anaplasma phagocytophilum* isolate G22 | AY281818 | *Ixodes ricinus* | Germany | 1 | I |
| *Anaplasma phagocytophilum* isolate 472 | AF478561 | *Capreolus capreolus* | Slovenia | 1 | I |
| *Ehrlichia phagocytophila* #99 | AF383227 | *Capreolus capreolus* | Switzerland | 1 | I |
| *Anaplasma phagocytophilum* isolate 70 | JN005748 | *Capreolus capreolus* | Poland | 1 | I |
| *Anaplasma phagocytophilum* isolate 832 | AF478553 | *Cervus elaphus* | Slovenia | 1 | I |
| *Ehrlichia phagocytophila* | U96730 | Sheep | Great Britain | 1 | I |
| *Ehrlichia phagocytophila* | U96729 | Goat | Great Britain | 1 | I |
| *Anaplasma phagocytophilum* strain c-D3160 | KM215266 | *Rupicapra rupicapra* | Slovenia | 1 | I |
| *Anaplasma phagocytophilum* strain tick-EU431 | KM215251 | *Ixodes ricinus* | Slovenia | 1 | I |
| *Anaplasma phagocytophilum* isolate 2 | AF548386 | Sheep | Norway | 1 | I |
| *Anaplasma phagocytophilum* strain c-D3155 | KM215265 | *Rupicapra rupicapra* | Slovenia | 1 | I |
| *Anaplasma phagocytophilum* isolate GC45 | HM057228 | *Ixodes ricinus* | Spain | 1 | I |
| *Anaplasma phagocytophilum* isolate 0511 | HM057225 | *Cervus elaphus* | Spain | 1 | I |
| *Anaplasma phagocytophilum* strain c-2829 | KM215264 | *Rupicapra rupicapra* | Slovenia | 1 | I |
| *Anaplasma phagocytophilum* isolate 09/78 | HM057232 | *Ixodes ricinus* | Rusia | 1 | I |
| *Anaplasma phagocytophilum* strain bear-9304 | KJ622308 | *Ursus arctos* | Slovenia | 1 | I |
| *Anaplasma phagocytophilum* isolate 474 | AF478563 | *Cervus elaphus* | Slovenia | 1 | I |
| *Anaplasma phagocytophilum* strain bear-9503 | KJ622307 | *Ursus arctos* | Slovenia | 1 | I |
| *Anaplasma phagocytophilum* isolate dog-7425 | EU381151 | Dog | Slovenia | 1 | I |
| *Anaplasma phagocytophilum* isolate G55 | AY281823 | *Ixodes ricinus* | Germany | 1 | I |
| *Anaplasma phagocytophilum* GroESL | EF392724 | *Ixodes ricinus* | Croatia | 1 | I |
| *Anaplasma phagocytophilum* isolate tick-40 | EU381152 | *Ixodes ricinus* | Slovenia | 1 | I |
| *Anaplasma phagocytophilum* strain tick-EU343 | KM215246 | *Ixodes ricinus* | Slovenia | 1 | I |
| *Anaplasma phagocytophilum* isolate 921 | AF478558 | *Capreolus capreolus* | Slovenia | 1 | I |
| *Anaplasma phagocytophilum* isolate 61g | HM057230 | *Ixodes ricinus* | Spain | 1 | I |
| *Anaplasma phagocytophilum* isolate 1 | AF548385 | Sheep | Norway | 1 | I |
| *Anaplasma phagocytophilum* isolate 09/71 | HM057231 | *Ixodes ricinus* | Spain | 1 | I |
| *Anaplasma phagocytophilum* strain c-708 | KM215263 | *Rupicapra rupicapra* | Slovenia | 1 | I |
| *Anaplasma phagocytophilum* isolate W271 | AY281844 | *Ixodes ricinus* | Germany | 1 | I |
| *Anaplasma phagocytophilum* strain tick-EU108 | KM215252 | *Ixodes ricinus* | Slovenia | 1 | I |
| *Anaplasma phagocytophilum* isolate I94 | AY281828 | *Ixodes ricinus* | Germany | 1 | I |
| *Ehrlichia phagocytophila* | AF202895 | *Ixodes ricinus* | Switzerland | 1 | I |
| *Anaplasma phagocytophilum* isolate 3C/2310/1 | HM057224 | *Cervus elaphus* | Spain | 1 | I |
| *Anaplasma phagocytophilum* isolate L6-9 | HM057233 | *Ixodes ricinus* | Russia | 1 | I |
| *Anaplasma phagocytophilum* isolate tick-43 | EU246959 | *Ixodes ricinu* | Slovenia | 1 | I |
| *Anaplasma phagocytophilum* isolate GC19 | HM057227 | *Ixodes ricinus* | Spain | 1 | I |
| *Anaplasma phagocytophilum* strain red-D3217 | KM215262 | *Cervus elaphus* | Slovenia | 1 | I |
| *Anaplasma phagocytophilum* isolate 473 | AF478562 | *Cervus elaphus* | Slovenia | 1 | I |
| *Anaplasma phagocytophilum* isolate 707 | AF478557 | *Cervus elaphus* | Slovenia | 1 | I |
| *Anaplasma phagocytophilum* strain red-D3009 | KM215261 | *Cervus elaphus* | Slovenia | 1 | I |
| *Anaplasma phagocytophilum* isolate 812 groESL | AF478552 | *Cervus elaphus* | Slovenia | 1 | I |
| *Anaplasma phagocytophilum* isolate dog-7414 | EU381150 | Dog | Slovenia | 1 | I |
| *Anaplasma phagocytophilum* | EU184703 | *Sus scrofa* | Slovenia | 1 | I |
| *Anaplasma phagocytophilum* isolate N6 | AY281849 | *Ixodes ricinus* | Germany | 1 | I |
| *Ehrlichia* sp. 'HGE agent' | AF033101 | Human | Slovenia | 1 | I |
| *Anaplasma phagocytophilum* | AF482760 | Horse | Germany | 1 | I |
| *Anaplasma phagocytophilum* strain Strong | AY529490 | Horse | Sweden | 1 | I |
| *Anaplasma phagocytophilum* isolate 163HFIRQ | KF383241 | *Ixodes ricinus* | Slovakia | 1 | I |
| *Anaplasma phagocytophilum* isolate 39FCIRQ | KF383239 | *Ixodes ricinus* | Slovakia | 1 | I |
| *Anaplasma phagocytophilum* isolate 187 | EU860089 | Sheep | France | 1 | I |
| *Anaplasma phagocytophilum* | KF836094 | Dog | Brazil | 1 | I |
| *Anaplasma phagocytophilum* strain Susy | AY529489 | Horse | Sweden | 1 | I |
| *Anaplasma phagocytophilum* strain Fordyce | EF647585 | Horse | USA | 1 | I |
| *Anaplasma phagocytophilum* | DQ680012 | Cat | USA | 1 | I |
| *Anaplasma phagocytophilum* | AY219849 | Dog | USA | 1 | I |
| *Ehrlichia equi* | AF173989 | *Ixodes pacificus* | USA | 1 | I |
| *Ehrlichia equi* | AF173988 | *Neotoma fuscipes* | USA | 1 | I |
| *Anaplasma phagocytophilum* clone KC15 | AY626252 | *Sigmodon hispidus* | USA | 1 | I |
| *Ehrlichia equi* isolate CAMAWI | AF172160 | Horse | USA | 1 | I |
| *Ehrlichia* sp. 'HGE agent' isolate CAHU-HGE2 | AF172159 | Human | USA | 1 | I |
| *Ehrlichia equi* isolate CASOLJ | AF172158 | Horse | USA | 1 | I |
| *Anaplasma phagocytophilum* strain GACTR12 | DQ088133 | *Sylvilagus floridanus* | USA | 1 | I |
| *Anaplasma phagocytophilum* isolate 151 | EU157921 | *Capreolus capreolus* | Poland | 1 | I |
| *Anaplasma phagocytophilum* strain ST-156 | DQ779567 | *Cervus elaphus* | Poland | 1 | I |
| *Anaplasma phagocytophilum* isolate 9B13 | KC800986 | *Alces alces* | Sweden | 1 | I |
| *Anaplasma phagocytophilum* isolate Nf_DU1_HW | JF494841 | *Neotoma fuscipes* | USA | 1 | I |
| *Anaplasma phagocytophilum* isolate Nf_1603_HV | JF494836 | *Neotoma fuscipes* | USA | 1 | I |
| *Anaplasma phagocytophilum* isolate Nf_1629_HC | JF494835 | *Neotoma fuscipes* | USA | 1 | I |
| *Anaplasma phagocytophilum* isolate Nf_1619_HC | JF494834 | *Neotoma fuscipes* | USA | 1 | I |
| *Anaplasma phagocytophilum* isolate Dog_CA | JF494833 | Dog | USA | 1 | I |
| *Ehrlichia* sp. 'HGE agent' | U72628 | Human | USA | 1 | I |
| *Anaplasma phagocytophilum* str. JM | CP006617 | Human | USA | 1 | I |
| *Anaplasma phagocytophilum* str. Dog2 | CP006618 | Dog | USA | 1 | I |
| *Anaplasma phagocytophilum* strain Webster | EU860090 | Ruminants | France | 1 | I |
| *Anaplasma phagocytophilum* isolate GV348 | MK341070 | *Ixodes ricinus* | Slovakia | 1 | I |
| *Anaplasma phagocytophilum* isolate 14DRS | KR092132 | *Sus scrofa* | Slovakia | 1 | I |
| *Anaplasma phagocytophilum* isolate J | KF312361 | *Ixodes ricinus* | Poland | 1 | I |
| *Anaplasma phagocytophilum* isolate G | KF312358 | *Ixodes ricinus* | Poland | 1 | I |
| *Anaplasma phagocytophilum* isolate F | KF312360 | *Ixodes ricinus* | Poland | 1 | I |
| *Anaplasma phagocytophilum* isolate E | KF312357 | *Ixodes ricinus* | Poland | 1 | I |
| *Anaplasma phagocytophilum* isolate I | KF312359 | *Ixodes ricinus* | Poland | 1 | I |
| *Anaplasma phagocytophilum* isolate H | KF312355 | *Ixodes ricinus* | Poland | 1 | I |
| *Anaplasma phagocytophilum* isolate Z15 | MW762533 | *Lepus europaeus* | Czech Republic | 1 | I |
| *Anaplasma phagocytophilum* isolate 16Pl | MG670108 | *Procyon lotor* | Poland | 1 | I |
| *Anaplasma phagocytophilum* strain ItalyIRH01241 | KF031388 | *Ixodes ricinus* | Italy | 1 | I |
| *Anaplasma phagocytophilum* isolate 2916 | EU860087 | Cow | France | 1 | I |
| *Anaplasma phagocytophilum* isolate 811 | AF478551 | *Capreolus capreolus* | Slovenia | 3 | II |
| *Anaplasma phagocytophilum* isolate 470 | AF478559 | *Capreolus capreolus* | Slovenia | 3 | II |
| *Anaplasma phagocytophilum* strain rod-1427 | KM215256 | *Capreolus capreolus* | Slovenia | 3 | II |
| *Anaplasma phagocytophilum* isolate D21 | AY281816 | *Ixodes ricinus* | Germany | 3 | II |
| *Anaplasma phagocytophilum* isolate 805 | AF478555 | *Capreolus capreolus* | Slovenia | 3 | II |
| *Anaplasma phagocytophilum* strain rod-1693 | KM215255 | *Capreolus capreolus* | Slovenia | 3 | II |
| *Anaplasma phagocytophilum* strain tick-EU136 | KM215250 | *Ixodes ricinus* | Slovenia | 3 | II |
| *Anaplasma phagocytophilum* isolate G26 | AY281820 | *Ixodes ricinus* | Germany | 3 | II |
| *Anaplasma phagocytophilum* isolate 794 | AF478556 | *Capreolus capreolus* | Slovenia | 3 | II |
| *Anaplasma phagocytophilum* strain tick-EU260 | KM215249 | *Ixodes ricinus* | Slovenia | 3 | II |
| *Anaplasma phagocytophilum* isolate A4 GroEL | AY220469 | *Capreolus capreolus* | Austria | 3 | II |
| *Anaplasma phagocytophilum* strain rod-1424 | KM215259 | *Capreolus capreolus* | Slovenia | 3 | II |
| *Anaplasma phagocytophilum* strain rod-1694 | KM215258 | *Capreolus capreolus* | Slovenia | 3 | II |
| *Anaplasma phagocytophilum* isolate 806 | AF478554 | *Capreolus capreolus* | Slovenia | 3 | II |
| *Anaplasma phagocytophilum* isolate A6 | AY220470 | *Ixodes ricinus* | Austria | 3 | II |
| *Anaplasma phagocytophilum* strain tick-EU322 | KM215247 | *Ixodes ricinus* | Slovenia | 3 | II |
| *Ehrlichia phagocytophila* #56 | AF383225 | *Capreolus capreolus* | Switzerland | 3 | II |
| *Anaplasma phagocytophilum* strain rod-1429 | KM215257 | *Capreolus capreolus* | Slovenia | 3 | II |
| *Anaplasma phagocytophilum* isolate 478 | AF478564 | *Capreolus capreolus* | Slovenia | 3 | II |
| *Anaplasma phagocytophilum* strain tick-EU329 | KM215248 | *Ixodes ricinus* | Slovenia | 3 | II |
| *Anaplasma phagocytophilum* strain rod-1691 | KM215254 | *Capreolus capreolus* | Slovenia | 3 | II |
| *Anaplasma phagocytophilum* isolate I63 | AY281825 | *Ixodes ricinus* | Germany | 3 | II |
| *Anaplasma phagocytophilum* strain rod-1686 | KM215253 | *Capreolus capreolus* | Slovenia | 3 | II |
| *Anaplasma phagocytophilum* isolate 47 | JN005747 | *Capreolus capreolus* | Poland | 3 | II |
| *Anaplasma phagocytophilum* isolate 09 | JN005743 | *Capreolus capreolus* | Poland | 3 | II |
| *Anaplasma phagocytophilum* strain Italy59_3 | KF031400 | *Ixodes ricinus* | Italy | 3 | II |
| *Anaplasma phagocytophilum* strain ItalyIRH018611 | KF031394 | *Ixodes ricinus* | Italy | 3 | II |
| *Anaplasma phagocytophilum* strain ItalyIRH012211 | KF031392 | *Ixodes ricinus* | Italy | 3 | II |
| *Anaplasma phagocytophilum* strain Italy25 | KF031382 | *Ixodes ricinus* | Italy | 3 | II |
| *Anaplasma phagocytophilum* strain Italy21b | KF031380 | *Ixodes ricinus* | Italy | 3 | II |
| *Anaplasma phagocytophilum* strain ST-128 | DQ779568 | *Capreolus capreolus* | Poland | 3 | II |
| *Anaplasma phagocytophilum* isolate S40 GroEL | KC800984 | *Alces alces* | Sweden | 3 | II |
| *Anaplasma phagocytophilum* isolate Italy737 | EU552920 | *Ixodes ricinus* | Italy | 3 | II |
| *Anaplasma phagocytophilum* isolate Italy738 | EU552918 | *Ixodes ricinus* | Italy | 3 | II |
| *Anaplasma phagocytophilum* isolate K | KF312356 | *Ixodes ricinus* | Poland | 3 | II |
| *Anaplasma phagocytophilum* isolate fricoe | JX082324 | *Ixodes ricinus* | Switzerland | 3 | II |
| *Anaplasma phagocytophilum* isolate erirub | JX082325 | *Ixodes ricinus* | Switzerland | 3 | II |
| Uncultured *Anaplasma* sp. clone Ip11-2 | JQ622144 | *Ixodes persulcatus* | Japan | 4 | II |
| *Anaplasma phagocytophilum* isolate Kh-395_Ip | MN989865 | *Ixodes persulcatus* | Russia | 4 | II |
| *Anaplasma phagocytophilum* isolate Kh-434_Ip | MN989863 | *Ixodes persulcatus* | Russia | 4 | II |
| *Anaplasma phagocytophilum* isolate Irk-Ip776 | HM366573 | *Ixodes persulcatus* | Russia | 4 | II |
| *Anaplasma phagocytophilum* isolate Irk-Ip820 | HM366574 | *Ixodes persulcatus* | Russia | 4 | II |
| *Anaplasma phagocytophilum* isolate Kh-chipmunk177 | HQ630619 | *Tamias sibiricus* | Russia | 4 | II |
| *Anaplasma phagocytophilum* isolate Irk-Ip662 | HM366572 | *Ixodes persulcatus* | Russia | 4 | II |
| *Anaplasma phagocytophilum* isolate Kh-Ip7 | HM366575 | *Ixodes persulcatus* | Russia | 4 | II |
| *Anaplasma phagocytophilum* isolate Kh-Ip80 | HM366576 | *Ixodes persulcatus* | Russia | 4 | II |
| *Anaplasma phagocytophilum* isolate Kh-Ip144 | HM366577 | *Ixodes persulcatus* | Russia | 4 | II |
| *Anaplasma phagocytophilum* isolate Kh-868_Tsib | MN989862 | *Tamias sibiricus* | Russia | 4 | II |
| *Anaplasma phagocytophilum* isolate RUS/Nov14-1682-Ipr/Ipv | MG182154 | *Ixodes persulcatus* | Russia | 4 | II |
| *Anaplasma phagocytophilum* isolate RUS/Nov14-1768-Ipv | KX980043 | *Ixodes pavlovskyi* | Russia | 4 | II |
| *Anaplasma phagocytophilum* isolate Nov-chipmunk1322 | HQ630618 | *Tamias sibiricus* | Russia | 4 | II |
| *Anaplasma phagocytophilum* isolate Sv-Ip854 | HM366567 | *Ixodes persulcatus* | Russia | 4 | II |
| *Anaplasma phagocytophilum* isolate Nov-Ip364 | HM366569 | *Ixodes persulcatus* | Russia | 4 | II |
| *Anaplasma phagocytophilum* isolate Omsk-373_Ip | MN701641 | *Ixodes persulcatus* | Russia | 4 | II |
| *Anaplasma phagocytophilum* clone Omsk-vole83 | KC583433 | *Myodes glareolus* | Russia | 4 | II |
| *Anaplasma phagocytophilum* isolate Nov-Ip355 | HM366568 | *Ixodes persulcatus* | Russia | 4 | II |
| *Anaplasma phagocytophilum* isolate Kh-Ip160 | HM366578 | *Ixodes persulcatus* | Russia | 4 | II |
| *Anaplasma phagocytophilum* strain AAIK2 GroES | KT220191 | *Apodemus agrarius* | South Korea | 4 | II |
| *Anaplasma phagocytophilum* strain AAIK3 | KT220192 | *Apodemus agrarius* | South Korea | 4 | II |
| *Anaplasma phagocytophilum* strain AAIK1 | KT192430 | *Apodemus agrarius* | South Korea | 4 | II |
| *Anaplasma phagocytophilum* isolate Hongdo-11-1 | JX219474 | *Ixodes nipponensis* | South Korea | 4 | II |
| *Anaplasma phagocytophilum* clone KWDTAPg | JQ086319 | *Haemaphysalis flava* | South Korea | 4 | II |
| *Anaplasma phagocytophilum* isolate KWDAPg | HM752098 | *Hydropotes inermis* | South Korea | 4 | II |
| *Anaplasma phagocytophilum* Yeyasu | LC334016 | Dog | Japan | 4 | II |
| *Anaplasma phagocytophilum* isolate gw1 | KJ677107 | Human | South Korea | 4 | II |
| *Anaplasma phagocytophilum* isolate D-SE-63 | KU519286 | Dog | South Korea | 4 | II |
| *Anaplasma phagocytophilum* isolate S-DD-20 | KU519284 | Cat | South Korea | 4 | II |
| *Anaplasma phagocytophilum* strain N6Bel | JX133177 | *Ixodes ricinus* | Switzerland | 3 | II |
| *Anaplasma phagocytophilum* strain ItalyIRH017411 | KF031393 | *Ixodes ricinus* | Italy | 7 | IV |
| *Anaplasma phagocytophilum* isolate turmer | JX082323 | *Ixodes ricinus* | Switzerland | 7 | IV |
| *Anaplasma phagocytophilum* strain Patagonia 5P | OP585578 | *Pudu puda* | Chile | 8 | V |
| *Anaplasma phagocytophilum* strain Patagonia 7P | OP585579 | *Pudu puda* | Chile | 8 | V |
| *Anaplasma phagocytophilum* strain Patagonia IS8 | OP585581 | *Ixodes stilesi* | Chile | 8 | V |
| *Anaplasma phagocytophilum* strain Patagonia IS17 | OP585582 | *Ixodes stilesi* | Chile | 8 | V |
| *Anaplasma phagocytophilum* strain Patagonia IS21 | OP585585 | *Ixodes stilesi* | Chile | 8 | V |
| *Anaplasma phagocytophilum* strain Patagonia 36S | OP585589 | *Pudu puda* | Chile | 8 | V |
| *Anaplasma phagocytophilum* strain Patagonia 24P | OP585590 | *Pudu puda* | Chile | 8 | V |
| *Anaplasma phagocytophilum* strain Patagonia IS25 | OP585588 | *Ixodes stilesi* | Chile | 8 | V |
| *Anaplasma phagocytophilum* strain Patagonia 8P | OP585580 | *Pudu puda* | Chile | 8 | V |
| *Anaplasma phagocytophilum* strain Patagonia IS20 | OP585586 | *Ixodes stilesi* | Chile | 8 | V |
| *Anaplasma phagocytophilum* strain Patagonia IS22 | OP585587 | *Ixodes stilesi* | Chile | 8 | V |
| *Anaplasma phagocytophilum* strain Patagonia IS18 | OP585583 | *Ixodes stilesi* | Chile | 8 | V |
| *Anaplasma phagocytophilum* strain Patagonia IS19 | OP585584 | *Ixodes stilesi* | Chile | 8 | V |
| ***Anaplasma* *phagocytophilum* strain Uruguay_S23IpN2_CUE** | **PX394611** | ***Ixodes fuscipes*** | **Uruguay** | **9** | **VI** |
| ***Anaplasma phagocytophilum* strain Uruguay_S32IpN14_CUE** | **PX394612** | ***Ixodes fuscipes*** | **Uruguay** | **9** | **VI** |
| ***Anaplasma* *phagocytophilum* strain Uruguay_S27IpN5_LUN** | **PX394613** | ***Ixodes fuscipes*** | **Uruguay** | **9** | **VI** |
| ***Anaplasma* *phagocytophilum* strain Uruguay_S28IpN13_LUN** | **PX394614** | ***Ixodes fuscipes*** | **Uruguay** | **9** | **VI** |
| *Anaplasma phagocytophilum* | AY279085 | Goat | Albania | Unassigned | Unassigned |
| *Anaplasma phagocytophilum* isolate 77HNIRQ | KF383240 | *Ixodes ricinus* | Slovakia | Unassigned | Unassigned |
| *Anaplasma phagocytophilum* isolate 5NBZIRQ | KF383238 | *Ixodes ricinus* | Slovakia | Unassigned | Unassigned |
| *Anaplasma phagocytophilum* isolate 42 | EU157920 | *Capreolus capreolus* | Poland | Unassigned | Unassigned |
| *Anaplasma platys* strain RP | EU516386 |  |  | Not applicable | Not applicable |
| *Anaplasma platys* strain WHBMXZ-126 | KX987394 | *Boophilus microplus* | China | Not applicable | Not applicable |
| *Anaplasma odocoilei* strain UMUM76 | JX876642 | *Odocoileus virginianus* |  | Not applicable | Not applicable |
| *Anaplasma marginale* isolate CNP_976_2 | KY305561 | *Syncerus caffer* | South Africa | Not applicable | Not applicable |
| *Anaplasma marginale* isolate AEP_1007_3 | KY305562 | *Syncerus caffer* | South Africa | Not applicable | Not applicable |
| *Anaplasma centrale* isolate Ac1_Ug_Ktd_2013_KR5_30h | KY523000 | Cattle | Uganda | Not applicable | Not applicable |
| *Anaplasma ovis* isolate OVI | AF441131 |  | South Africa | Not applicable | Not applicable |
| *Anaplasma ovis* isolate 76 | FJ460441 |  | Cyprus | Not applicable | Not applicable |
| *Anaplasma capra* strain HLJ-14 | KM206275 | Human | China | Not applicable | Not applicable |
| *Anaplasma capra* isolate Hstaji200 | MZ222248 | *Equus hemionus onager* | Iran | Not applicable | Not applicable |
| *Ehrlichia ruminantium* strain Kumm2 | DQ647013 |  | South Africa | Not applicable | Not applicable |
